# Supplementary material for: Randomness as a driver of inactivity in social groups
Source: PLoS Comput Biol. 2024 Dec 17;20(12):e1012668. doi: 10.1371/journal.pcbi.1012668 (PMC11651616; doi:10.1371/journal.pcbi.1012668)
Supplement: S1 Text — (RTF) [file pcbi.1012668.s001.rtf]

import java.io.FileOutputStream;import java.io.IOException;import java.io.PrintStream;import java.util.Arrays;import java.util.Random;public class DOL{	public static void main(String[ ]arguments)	{	try {		FileOutputStream fout1 = new FileOutputStream("BestEfficiency.txt"); PrintStream myOutput1 = new PrintStream(fout1); 		FileOutputStream fout2 = new FileOutputStream("DynCostsPerf.txt"); PrintStream myOutput2 = new PrintStream(fout2);		int colSize = 500;		int production_costs = 100; // (arbitrary unit) invariant with colony size        		double maintenance_costs_active = 0; // invariant with colony size        		double maintenance_costs_inactive = 0; // invariant with colony size	      		double daily_costs = 0;        		int Nsimul =  1000;// number of simulations        		int Nactive=0;       		int Ninactive=0;		double colEfficiency=0;		double tempNactive=0;		double tempcumul_perf=0;		double tempdaily_costs=0;						for (int groupSize=1; groupSize<=3;groupSize++ )		{		if (groupSize==1) { colSize=50; Nsimul=1000;}		if (groupSize==2) { colSize=500; Nsimul=1000;}		if (groupSize==3) { colSize=5000; Nsimul=100;}				for (int simulation=1; simulation<=Nsimul;simulation++ )	      		{			for (int theoDistrib=1; theoDistrib<=5;theoDistrib++ ) // 5 different random distributions 			{					for (double ratio_costs_inactive_active=0.1; ratio_costs_inactive_active<=1.01; ratio_costs_inactive_active +=0.05)// ratio of maintenance costs between inactive and active workers				{								for (double ratio_maintenance_production=0.0; ratio_maintenance_production<10.1;ratio_maintenance_production+=0.1)//ratio of production to maintenance costs 					{											int minInd=10; // minimal colony size						int incrementSize=5; // increase in colony size																int counter=0; // to store results, one row for each increment in group size						double [][] matrice =new double[(int)(1+(colSize-minInd)/incrementSize)][4];											 // individual efficiency (random values between 0 and 1)				         		double [] distrib_worker_efficiency =new double[colSize];		        				         		// truncated normal distribution				         		double meanEfficiency=0;						 double sdEfficiency=0;					 				         		 // right skewed distribution				         		 double skewness=4;			         								 if(theoDistrib==1)				        		RightSkewedDist (distrib_worker_efficiency,colSize,skewness);			          			 		         // random distribution				          			 		         if(theoDistrib==2)  			 		         {      				 		       	meanEfficiency= 0.5; sdEfficiency= 0.2;					       	GenerateRdmNorm(distrib_worker_efficiency,colSize, meanEfficiency, sdEfficiency);			         			}			       			          			         			// bimodal normal distribution			          			double PropPop=0.5;			          			double meanEfficiency1= 0.1; double sdEfficiency1= 0.2;			          			double meanEfficiency2= 0.9; double sdEfficiency2= 0.2;        			         			if(theoDistrib==3)     						GenerateRdm_Bi_Norm (distrib_worker_efficiency,colSize,  PropPop,  meanEfficiency1,  sdEfficiency1,  meanEfficiency2,  sdEfficiency2) ;			         			         			           		         			          			if(theoDistrib==4)  // uniform distribution			        			GenerateRdmUnif (distrib_worker_efficiency,colSize);			          			          			if(theoDistrib==5)			        			LeftSkewedDist (distrib_worker_efficiency,colSize,skewness);			 			         			 // individual performance			          			double [] distrib_worker_performance =new double[colSize];			    					            			         			for (int j=0; j<colSize; j++) 			           		 {			            	 		distrib_worker_performance[j] = distrib_worker_efficiency[j];			           		 }			            			            		Arrays.sort(distrib_worker_performance);//individual performance sorted in ascending order			            			          											for (int NIndAct=minInd; NIndAct<=colSize; NIndAct+=incrementSize )						{												Nactive = NIndAct;						Ninactive = colSize-Nactive;										     		 // daily costs :maintenance costs of all workers and investment to produce new workers (i.e. colony size)				         		maintenance_costs_active = ratio_maintenance_production * production_costs ; 				          		maintenance_costs_inactive = ratio_costs_inactive_active * ratio_maintenance_production * production_costs ; 	 				        		 				         		daily_costs = (Nactive * maintenance_costs_active) + (Ninactive * maintenance_costs_inactive) + ((colSize * production_costs));			      				          			           		double cumul_perf=0;			           		for (int j = 0 ; j< Nactive ; j++) 			           		{			            		cumul_perf=cumul_perf+distrib_worker_performance[(colSize-1)-j];			            		}					           	matrice[counter][0]=cumul_perf/daily_costs;//store each value of colony efficiency for each number of active individuals				           	matrice[counter][1]=Nactive;				            	matrice[counter][2]=cumul_perf;				            	matrice[counter][3]=daily_costs;				            	counter=counter+1;				            			       				            	// to store a subset of the results for plotting results (middle column of Figure 1)						if(ratio_costs_inactive_active>0.199 & ratio_costs_inactive_active<0.201 & ratio_maintenance_production>1.999 & ratio_maintenance_production<2.001)						{						myOutput2.println(theoDistrib+"\t"+simulation+"\t"+colSize+"\t"+ratio_costs_inactive_active+"\t"+ratio_maintenance_production+"\t"+Nactive+"\t"+cumul_perf +"\t"+daily_costs);						}					}					//identify the highest efficiency (ie cumul_perf/daily_costs)					colEfficiency=matrice[0][0];					tempNactive=matrice[0][1];					tempcumul_perf=matrice[0][2];					tempdaily_costs=matrice[0][3];										for (int i = 0 ; i< counter ; i++) 		          			{						if(matrice[i][0]>colEfficiency)						{							colEfficiency=matrice[i][0];							tempNactive=matrice[i][1];							tempcumul_perf= matrice[i][2];							tempdaily_costs=matrice[i][3];						}		           		}												myOutput1.println(theoDistrib+"\t"+simulation+"\t"+colSize+"\t"+ratio_costs_inactive_active+"\t"+ratio_maintenance_production+"\t"+colEfficiency+"\t"+tempcumul_perf +"\t"+tempdaily_costs+"\t"+ tempNactive);				}			}					}		}}		}// try ends herecatch (IOException e) {	System.err.println("Error: " + e);System.exit(1);} }	// 5 random generators		 static void GenerateRdmUnif (double RndN [], int Nworkers) 	{	      Random rd = new Random(); // creating Random object	     	      	      for (int i = 0; i < Nworkers; i++) 	      {	    	 do {	    		 RndN[i] = rd.nextDouble(); // storing random integers in an array	    	 }	    	 while (RndN[i]  > 1 || RndN[i]  < 0);	      }	   }			 static void GenerateRdmNorm (double RndN [], int Nworkers, double meanEfficiency, double sdEfficiency) 	{	      Random rd = new Random(); // creating Random object	     	      	      for (int i = 0; i < Nworkers; i++) 	      {	    	 do {	    		 RndN[i] = (rd.nextGaussian()*sdEfficiency) + meanEfficiency; // storing random integers in an array	    	 }	    	 while (RndN[i]  > 1 || RndN[i]  < 0);	      }	   }	 	 static void GenerateRdm_Bi_Norm (double RndN [], int Nworkers, double PropNorm, double meanEfficiency1, double sdEfficiency1,  double meanEfficiency2, double sdEfficiency2) 		{		      Random rd = new Random(); 		      int Nworkers1= (int) (Nworkers * PropNorm); // determine number of individuals belonging to population 1		   		      		      for (int i = 0; i < Nworkers1; i++) 		      {		    	 do {		    		 RndN[i] = (rd.nextGaussian()*sdEfficiency1) + meanEfficiency1; // storing random integers in an array		    	 }		    	 while (RndN[i]  > 1 || RndN[i]  < 0);		      }		      		      		      		      for (int i = Nworkers1 ; i <Nworkers; i++) 		      {		    	 do {		    		 RndN[i] = (rd.nextGaussian()*sdEfficiency2) + meanEfficiency2; // storing random integers in an array		    	 }		    	 while (RndN[i]  >= 1 || RndN[i]  < 0);		      }		   }		 	 static void RightSkewedDist (double RndN [], int Nworkers, double skewness) 		{		      Random rd = new Random();		     		      		      for (int i = 0; i < Nworkers; i++) 		      {		    	 do {		             double u = rd.nextDouble(); // Uniform random variable between 0 and 1		              RndN[i] = 1-Math.pow(u, 1.0 / skewness);		    	 }		    	 while (RndN[i]  > 1 || RndN[i]  < 0);		      }		   }	 	 static void LeftSkewedDist (double RndN [], int Nworkers, double skewness) 		{		      Random rd = new Random(); 		      for (int i = 0; i < Nworkers; i++) 		      {		    	 do {		             double u = rd.nextDouble(); // Uniform random variable between 0 and 1		              RndN[i] = Math.pow(u, 1.0 / skewness);		    	 }		    	 while (RndN[i]  > 1 || RndN[i]  < 0);		      }		   }	 }
