# Supplementary figures and images for: Randomness as a driver of inactivity in social groups
Source: PLoS Comput Biol. 2024 Dec 17;20(12):e1012668. doi: 10.1371/journal.pcbi.1012668 (PMC11651616; doi:10.1371/journal.pcbi.1012668)

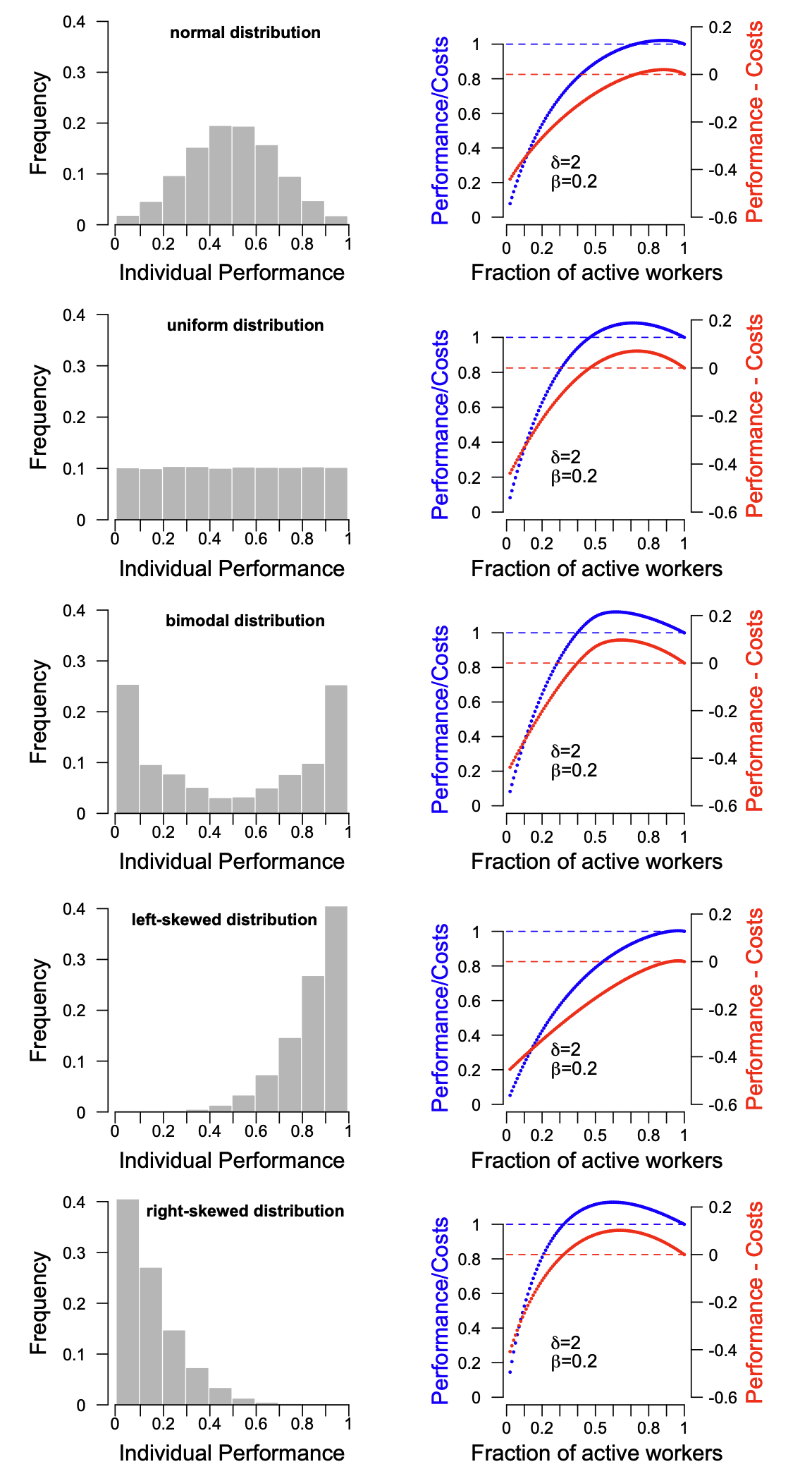

Supplement: S1 Fig — Colony efficiency as a function of the proportion of active workers. Left: Histogram of individual task performance for five different random distributions. Right: colony efficiency as a function of the proportion of active workers for values of δ = 2 and β = 0.2. Blue lines: efficiency measured as the ratio of performance to cost (as in Fig 1); red lines: efficiency measured as the difference between performance and cost. See Fig 1 for details. (TIF) [file pcbi.1012668.s002.tif]

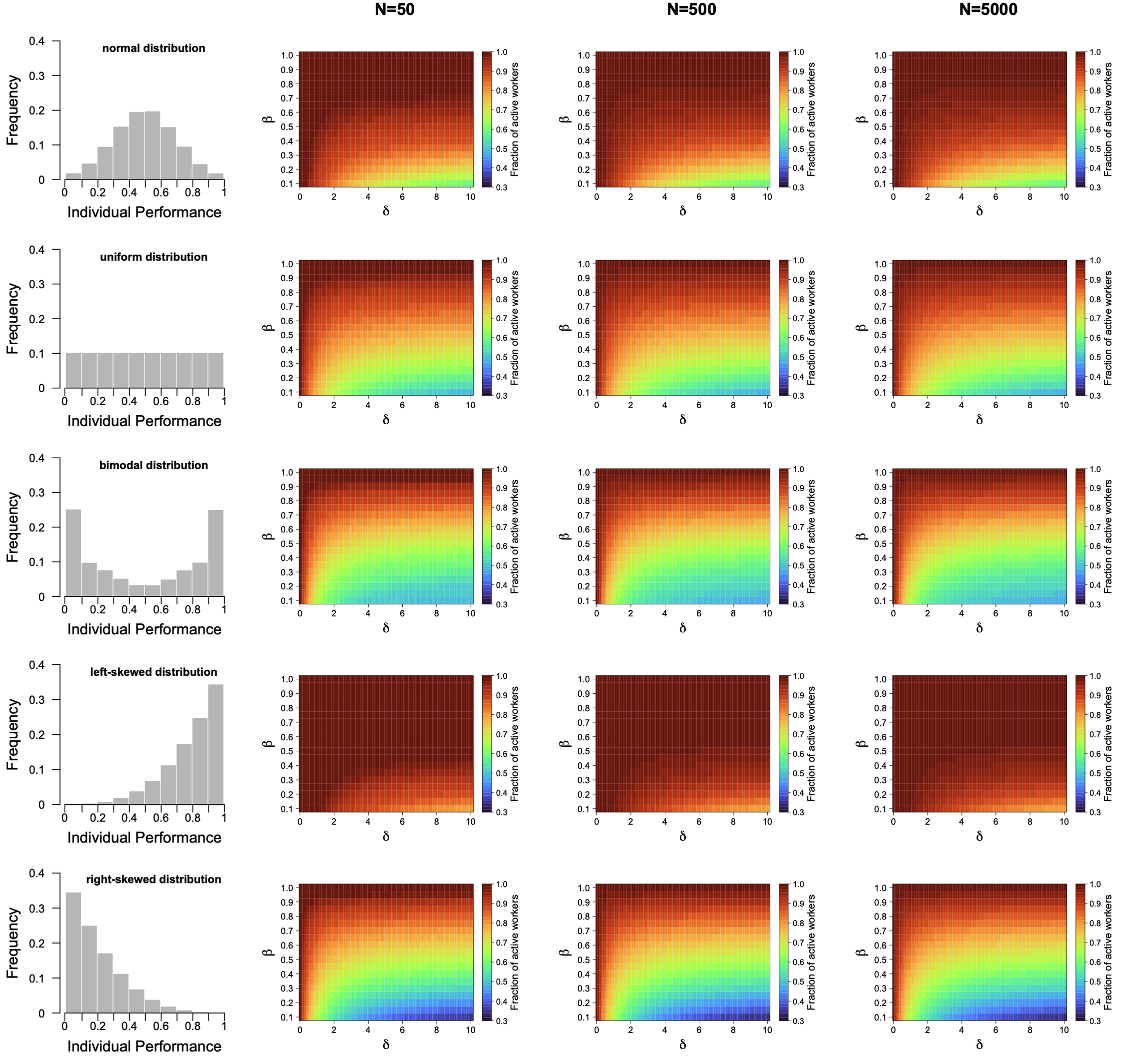

Supplement: S2 Fig — Colony efficiency as a function of the proportion of active workers for different colony size (50, 500, 5000 workers). Left: Histogram of individual task performances for five different random distributions. One thousand replicates were simulated for colonies of 50 and 500 workers and 100 replicates for colonies of 5000 workers. See Fig 1 for details. (TIF) [file pcbi.1012668.s003.tif]
