# Supplementary material for: Randomness as a driver of inactivity in social groups
Source: PLoS Comput Biol. 2024 Dec 17;20(12):e1012668. doi: 10.1371/journal.pcbi.1012668 (PMC11651616; doi:10.1371/journal.pcbi.1012668)
Supplement: Attachment — Submitted filename: PCOMPBIOL-D-24-00988.docx [file pcbi.1012668.s004.docx]

This paper addresses a question frequently discussed in the literature: the level of inactivity in insect societies. Various hypotheses have been proposed to explain this inactivity. In this theoretical paper, the authors propose a new and original hypothesis, based on the stochasticity of behavior and the production and maintenance costs of individuals.

It's a real pleasure to review this excellent paper presenting testable outcomes of a simple and parsimonious model.

I have no major comments, just a few minor suggestions and I strongly recommend to publish this article

Introduction

Line 50: “*If worker efficiency varies, a potential optimal strategy from a colony perspective might involve producing a large number of individuals*”. See my suggestions “analyzing the model”

Results and discussion

Lines 67 – 76 These conclusions are very interesting because these (qualitative) results are independent of the distribution of individual performances. The authors must insist on this result.

Model

Line 198 “ *The active individuals selected were those with the highest individual performance (for example, if 50 individuals were selected as active, we evaluated the colony's performance by aggregating the individual performances of the 50 best-performing workers)*”

I understand this rule (based on competition between workers?), but it would be useful for the reader to justify this choice.

Analyzing the model

Here are a few suggestions (that are not very time-consuming).

Why not add an analysis of the model by varying the colony size. If this won't affect the mean values, what about the variance? (variance decreases with colony size; =1/colony size?) (see line 50).

Are the model's predictions robust? For example, if efficiency is the difference between performance and costs, does this difference also show a maximum as a function of the fraction of active workers?

I have no problem with the tested distributions. However, these distributions have different mean values and variances (and other moments). It would be interesting to better identify the role of the mean and variance (to limit ourselves to the first two moments). For example, compare normal distribution with the same mean but with different variances.

Without criticizing the use of simulations, some of the results can easily (and quickly) be obtained analytically. For example, in the case of the uniform distribution (individual performance between 0 and 1), the mean performance

$$\sim\frac{0.5(2a-a^{2})}{1+\delta\beta+a\delta(1-\beta)};a=fraction of active workers$$

$$a that maximize the mean performance=-0.5U+0.5(U^{2}+4U)^{0.5}$$

$$U=\frac{2\left( 1+\delta\beta\right)}{\delta\left( 1-\beta\right)}$$
