# Supplementary material for: Randomness as a driver of inactivity in social groups
Source: PLoS Comput Biol. 2024 Dec 17;20(12):e1012668. doi: 10.1371/journal.pcbi.1012668 (PMC11651616; doi:10.1371/journal.pcbi.1012668)
Supplement: Attachment — Submitted filename: Responses to reviews R1.docx [file pcbi.1012668.s005.docx]

Dear Editor,

Thank you for your attention to our manuscript. We also sincerely thank the reviewers for their careful reading of our manuscript. We are pleased that they both recognised the originality and potential interest of our study. We appreciated the relevance and constructive nature of their comments, which helped to significantly improve the quality of our manuscript. In particular, we agree that the results should have been discussed more fully in the original version, which is now the case in the revised version. We have also included two additional figures (Figures S1, S2) to explore the model in line with the reviewers' comments. A full list of changes and detailed responses to comments follows (line numbers refer to the highlighted revised version of the manuscript).

**Reviewer 1**

This paper addresses a question frequently discussed in the literature: the level of inactivity in insect societies. Various hypotheses have been proposed to explain this inactivity. In this theoretical paper, the authors propose a new and original hypothesis, based on the stochasticity of behavior and the production and maintenance costs of individuals. It's a real pleasure to review this excellent paper presenting testable outcomes of a simple and parsimonious model. I have no major comments, just a few minor suggestions and I strongly recommend to publish this article.

- We sincerely thank Reviewer 1 for the positive evaluation of our manuscript.

Introduction

Line 50: “If worker efficiency varies, a potential optimal strategy from a colony perspective might involve producing a large number of individuals”. See my suggestions “analyzing the model”

- This revised version includes an exploration of the increase in group size on colony performance (lines 136-141, Figure S2).

Results and discussion

Lines 67 – 76 These conclusions are very interesting because these (qualitative) results are independent of the distribution of individual performances. The authors must insist on this result.

- We are now putting more emphasis on these findings (lines 110-111).

Model

Line 198 “The active individuals selected were those with the highest individual performance (for example, if 50 individuals were selected as active, we evaluated the colony's performance by aggregating the individual performances of the 50 best-performing workers)”

I understand this rule (based on competition between workers?), but it would be useful for the reader to justify this choice.

- We now justify this choice (lines 99-102).

Analyzing the model

Here are a few suggestions (that are not very time-consuming).

Why not add an analysis of the model by varying the colony size. If this won't affect the mean values, what about the variance? (variance decreases with colony size; =1/colony size?) (see line 50).

- We now present the results of the model for two additional colony sizes (50 and 5000 workers). The results are presented in Figure S2 and in the main text (lines 136-138).

Are the model's predictions robust? For example, if efficiency is the difference between performance and costs, does this difference also show a maximum as a function of the fraction of active workers?

- We thank Reviewer 1 for this suggestion. We now present a figure (Figure S1) in which we measure efficiency as the ratio between performance and cost, as well as the difference between performance and cost, based on the recommendation (Lines 249-250). The analysis shows that both methods give similar qualitative results. This indicates that our conclusions are robust, which is indicated in the text (Lines 138-141).

I have no problem with the tested distributions. However, these distributions have different mean values and variances (and other moments). It would be interesting to better identify the role of the mean and variance (to limit ourselves to the first two moments). For example, compare normal distribution with the same mean but with different variances.

- We agree with Reviewer 1. Three (i.e. normal, uniform and bimodal, Figure 1) of the five random distributions tested had the same mean but different variance. However, this point was not addressed in the manuscript. We now explicitly mention this aspect in the text (lines 111-114). We have also changed the order of presentation of the random distributions in Figure 1 to make this point easier to capture.

Without criticizing the use of simulations, some of the results can easily (and quickly) be obtained analytically. For example, in the case of the uniform distribution (individual performance between 0 and 1), the mean performance

$$\sim\frac{0.5(2a-a^{2})}{1+\delta\beta+a\delta(1-\beta)};a=fraction of active workers$$

$$a that maximize the mean performance=-0.5U+0.5(U^{2}+4U)^{0.5}$$

$$U=\frac{2\left( 1+\delta\beta\right)}{\delta\left( 1-\beta\right)}$$

- We are pleased that a different approach has led to similar conclusions, which further strengthens the relevance of our results.

**Reviewer #2**

In this manuscript, authors develop a model to examine worker inactivity in social insects like ants and bees. They support the idea that colonies generate many individuals to recruit the most efficient workers, despite the cost of maintaining inactive ones. Inactivity is suggested as a by-product of random processes “associated with the generation of individuals whose performance levels cannot be controlled”. The manuscript is well written, and the model comprehensively presented. I am not a theoretician, and therefore I can’t evaluate the technical details of the model. Instead, I have focused my review on the framing of the manuscript and on its broader contributions to the field. I am very positive about it but I think it needs quite a bit of work on presenting the current state of knowledge and the existing gaps. I hope that is still a helpful and constructive review.

- We thank Reviewer 2 for the positive evaluations and constructive comments, which helped improve the manuscript. We acknowledge that we could have better framed our manuscript and discussed our results. We believe that this revised version corrects the gaps identified by Reviewer 2.

The literature on the concept of redundancy in eusocial insect societies is quite rich, see for example Pedroso (2021). This paper is not discussed in the manuscript, despite it explores whether “redundancy can evolve as a by-product of selection when group members have to opt whether to cooperate or not while being blind to the strategies of others”. Pedroso’s work views the concept of redundancy through the lens of cooperation, and this perspective is overlooked in the current manuscript. Broadly, empirical tests and theoretical models have been employed to explore why there are inactive workers in animal societies. However, I believe that the manuscript doesn’t discuss the existing knowledge and developing hypotheses on the topic. This lack of background information doesn’t help the reader understand the new perspective that the manuscript contributes, where it agrees, and where it disagrees with the literature. I think that to improve this authors need to do literature searches, study their findings and re-write the introduction and discussion.

- We now provide more details on the introduction (Lines 42-50, 51-58) We have extended the discussion to talk about redundancy and how it relates to our results, in particular by commenting on the hypothesis in an article (Pedroso 2021) pointed out by Reviewer 2 (lines 184-193).

Another point I have is that beyond the production and maintenance costs of active and inactive workers, maintaining a large group size can also imply a broad range of extra costs (Krause & Ruxton, 2002), such as an increased risk for the spread of diseases or even higher inequality in the division of labour among group members (Rotics & Clutton-Brock, 2021). Authors should discuss additional costs associated with group size, as well as types of social organization, such as multilevel societies, which buffer those costs by allowing groups to associate when needed without maintaining a large group size at all times [see Camerlenghi & Papageorgiou (2024)].

- As we do not discuss the link between group size and the level of inactivity, as this would be beyond the scope of this paper, we feel it is not necessary to comment on the costs associated with increasing colony size.

L39 Why “mixed” support? Take space and time to explain the differences in these studies. I think this paragraph needs to be unpacked, expanded and explain what’s the new perspective that the current manuscript contributes, where it agrees and where it disagrees with the literature.

- This is now explained in more details (Lines 42-49).

L50-51 Why to assume that a larger number of workers implies higher variation? This has to be supported with references or simulations.

- Let's take an example: if we take a die and assume that the value obtained is equal to individual efficiency. By rolling the die several times, we increase the probability of obtaining a value of 6 at least once. Therefore, increasing group size increases the probability of obtaining *highly* efficient individuals. The sentence has been modified for clarity (Line 68).

L85 “This type” is unclear.

- This has been reworded (Line 128).

L100 This (Grüter et al., 2012) would be an interesting study to discuss here. I think the manuscript needs enrichment in terms of biological examples and animal species stories. PLOS Computational Biology is read by a wide audience that isn’t necessary familiar with the theory inspired by eusocial insects, therefore expanding the introduction and discussion in this direction would make the text more engaging and relevant for the readers of the journal.

- We now provide more biological examples both in the introduction and discussion (Lines 45-50, 61-62, 74-78, 144-147, 152-154, 174-175, 199-200).

L125-135 The flow in this part isn’t great and it repeats many points of the introduction. I think what would be very interesting to discuss is how the findings of this manuscript link/complement other models and empirical studies on the concept of redundancy in animal societies. Are there mutually exclusive hypotheses or the manuscript provides an additional explanation?

- The discussion has been amended to take account of this point, in particular to address the issue of redundancy (lines 184-193).

L136 Give ecological examples on what these functions could be.

- Examples of these functions are now provided (lines 198-200).

L135-139 This feels like repeating what was said before in the manuscript.

- This last section of the discussion has been modified.

L150 also the space they occupy in a shelter?

- It is not clear to us what the Reviewer is referring to here.

L196-197 I would discuss this assumption further because in eusocial insect societies with high levels of task specialisation and division of labour, body size can vary greatly and therefore maintenance costs across individuals may also vary.

- This aspect is now discussed in the main text (Lines 157-163).

L189 Why 500? What could be the effect of group size on all this? Discussion is missing.

- We now consider two additional group size (50 and 5,000 workers) (see Figure S2, Lines 136-138)

References

Camerlenghi, E., & Papageorgiou, D. (2024). Multilevel societies: different tasks at different social levels. EcoEvoRxiv. https://doi.org/https://doi.org/10.32942/X27C90

Grüter, C., Menezes, C., Imperatriz-Fonseca, V. L., & Ratnieks, F. L. W. (2012). A morphologically specialized soldier caste improves colony defense in a neotropical eusocial bee. Proceedings of the National Academy of Sciences, 109(4), 1182–1186. https://doi.org/10.1073/pnas.1113398109

Krause, J., & Ruxton, G. (2002). Living in groups. Oxford University Press.

Pedroso, M. (2021). Blind Cooperation: The Evolution of Redundancy via Ignorance. The British Journal for the Philosophy of Science, 72(3), 701–715. https://doi.org/10.1093/bjps/axz022

Rotics, S., & Clutton-Brock, T. (2021). Group size increases inequality in cooperative behaviour. Proceedings of the Royal Society B: Biological Sciences, 288(1945). https://doi.org/10.1098/rspb.2020.2104
