# Supplementary material for: Randomness as a driver of inactivity in social groups
Source: PLoS Comput Biol. 2024 Dec 17;20(12):e1012668. doi: 10.1371/journal.pcbi.1012668 (PMC11651616; doi:10.1371/journal.pcbi.1012668)
Supplement: Attachment — Submitted filename: Responses to reviews R2.docx [file pcbi.1012668.s006.docx]

We thank both reviewers for their careful consideration of our manuscript. We are pleased that they appreciated that our revised version has correctly addressed their queries. Reviewer 2 regretted that her/his comments regarding costs and group size had not been taken into account in our revisions. We now address this aspect briefly and also mention multi-leveled societies, as suggested. However, we would like to reiterate that we consider these points to be secondary, as our main focus is the relationship between maintenance costs and production costs.

**Reviewer 1**

I highly recommend this article for publication. The authors have largely taken my suggestions into account. In particular, they have included new results/analyses (concerning the influence of the size of the colony, criteria of efficiency). I would like congratulate the authors for their stimulating contribution. Moreover I suggest that they continue to exploit this model in future theoretical and experimental works.

We would like to thank Reviewer 1 for this evaluation and for her/his suggestions on the first version, which led to substantial improvements in the manuscript.

**Reviewer 2**

The authors have thoroughly revised their manuscript, but I think that one of my main comments was not addressed, with insufficient reasoning:

Reviewer 2, previous round: Another point I have is that beyond the production and maintenance costs of active and inactive workers, maintaining a large group size can also imply a broad range of extra costs (Krause & Ruxton, 2002), such as an increased risk for the spread of diseases or even higher inequality in the division of labour among group members (Rotics & Clutton-Brock, 2021). Authors should discuss additional costs associated with group size, as well as types of social organization, such as multilevel societies, which buffer those costs by allowing groups to associate when needed without maintaining a large group size at all times [see Camerlenghi & Papageorgiou (2024)].

Authors, previous round: As we do not discuss the link between group size and the level of inactivity, as this would be beyond the scope of this paper, we feel it is not necessary to comment on the costs associated with increasing colony size.

Reviewer 2, current round: Especially now that authors have run their simulations on three different group sizes, this discussion is missing even more than before. Inactive workers imply a larger group size (B) than that of a group with the same number of active workers but no inactive ones (A). In group B the cost of maintenance of inactive workers is larger than A, but there are multiple other costs that are not discussed in this manuscript, restrict group size in nature and are of critical importance, even if they aren’t examined through simulations here. In the previous round I mentioned only a few examples, but the list is longer. The example of the multilevel societies is also relevant as it shows how extra group members can be used when it’s needed, showing thus an alternative to the evolution of inactivity. I believe that a paragraph on these matters can be added in the discussion and would make the manuscript even more appealing.

We have added a section to the discussion dealing with these issues (lines 142-147).

Below I list line numbers on the document with track changes. I found quite a few typos and I believe that the manuscript should be read very carefully before resubmission.

L2 “organised” is quite vague, as most animal societies present some kind of organisation. For definition of social organisation see Kappeler 2019 in Behavioural Ecology.

The term ‘organised’ has been replaced by ‘structured’.

L22 “there are no clear explanations” or “there is no clear explanation”

done

L79 “than” should be deleted.

done

L184 “might also be true” instead of “might be also true”

done

L192 I am not sure about reference 33. Birch is a philosopher, and this paper is not a systematic review but a philosophical perspective. There are plenty of empirical studies on redundancy in biological systems that could be cited.

We have added two references.
